# An In Vitro and In Silico Study of Antioxidant Properties of Curcuminoid *N*-alkylpyridinium Salts: Initial Assessment of Their Antitumoral Properties

**DOI:** 10.3390/antiox11061104

**Published:** 2022-06-01

**Authors:** Oscar Forero-Doria, Luis Guzmán, Felipe Jiménez-Aspee, Javier Echeverría, Sergio Wehinger, Claudio Valenzuela, Ramiro Araya-Maturana, Maximiliano Martínez-Cifuentes

**Affiliations:** 1 Departamento de Ciencias Básicas, Facultad de Ciencias, Universidad Santo Tomás, Avenida Carlos Schorr 255, Talca 3460000, Chile; oforero@santotomas.cl; 2 Departamento de Bioquímica Clínica e Inmunohematología, Facultad de Ciencias de la Salud, Universidad de Talca, P.O. Box 747, Talca 3460000, Chile; lguzman@utalca.cl; 3 Institute of Nutritional Sciences, Department of Food Biofunctionality, University of Hohenheim, Garbenstrasse 28, 70599 Stuttgart, Germany; felipe.jimenez@nutres.de; 4 Departamento de Ciencias del Ambiente, Facultad de Química y Biología, Universidad de Santiago de Chile, Santiago 9170022, Chile; javier.echeverriam@usach.cl; 5 Thrombosis Research Center, Department of Clinical Biochemistry and Immunohematology, Faculty of Health Sciences, Medical Technology School, Universidad de Talca, Talca 3460000, Chile; snunez@utalca.cl; 6 Center for Medical Research, School of Medicine, University of Talca, Talca 3460000, Chile; cvalenzuela@utalca.cl; 7 Instituto de Química de Recursos Naturales, Universidad de Talca, P.O. Box 747, Talca 3460000, Chile; 8 MIBI: Interdisciplinary Group on Mitochondrial Targeting and Bioenergetics, Universidad de Talca, P.O. Box 747, Talca 3460000, Chile; 9 Departamento de Química Orgánica, Facultad de Ciencias Químicas, Universidad de Concepción, Edmundo Larenas 129, Concepción 4070371, Chile

**Keywords:** pyridinium salts, curcuminoids, antioxidants, Pabon reaction, cancer

## Abstract

In this work, we report the synthesis of curcuminoids with ionic liquid characteristics, obtained by incorporating alkyl-substituted pyridinium moiety rather than one phenyl group through a two-step process. The antioxidant capacity of the obtained compounds was evaluated in vitro by 1,1-diphenyl-picrylhydrazyl (DPPH) free radical scavenging and ferric reducing antioxidant power (FRAP) assays, showing that some derivatives are more potent than curcumin. Pyridine curcuminoids (group 4) and curcuminoid *N*-alkylpyridinium salts with two methoxyl groups in the phenyl ring (group 7), presented the best antioxidant capacity. The experimental results were rationalized by density functional theory (DFT) calculations of the bond dissociation enthalpy (BDE) for O–H in each compound. The computational calculations allowed for insight into the structural–antioxidant properties relationship in these series of compounds. BDEs, obtained in the gas phase and water, showed a notable impact of water solvation on the stabilization of some radicals. The lower values of BDEs in the water solution correspond to the structurally related compounds curcuminoid-pyridine **4c** and curcuminoid pyridinium salt **7a**, which is consistent with the experimental results. Additionally, an assessment of cell viability and cell migration assays was performed for human colon cancer (HT29), human breast cancer (MCF7) cells, in addition to NIH3T3 murine fibroblast, as a model of non-cancer cell type. These compounds mainly cause inhibition of the cell migration observed in MCF7 cancer cells without affecting the non-tumoral NIH3T3 cell line: Neither in viability nor in migration.

## 1. Introduction

**Figure 1:**
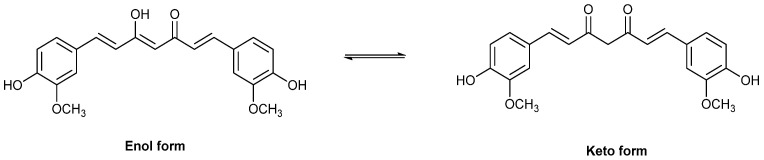
Structure of curcumin (enol and keto forms).

Curcumin (diferuloylmethane or 1,7-bis(4-hydroxy-3-methoxyphenyl)-1,6-heptadiene-3,5-dione) (Figure 1) has attracted considerable interest due to its diverse biological activities, which include antioxidant, antitumoral, antimicrobial, anti-inflammatory, antihepatotoxic, antihyperlipidemic, antiviral, and anti-Alzheimer’s disease. However, its poor solubility and poor bioavailability during oral administration result in limited clinical applicability [1]. Different strategies have been used to improve it, including encapsulating or incorporating curcumin in a nanoparticle or microparticle drug delivery system, synthesizing more stable curcumin analogs that resist metabolism while retaining the pharmacological properties of curcumin, adding another natural product that has bio-enhancing properties to curcumin or a combination of two of these strategies [2]. Compared with native curcumin, micellar curcumin and curcumin-γ-cyclodextrin complexes exhibit greater bioavailability in humans, indicating that the post-digestive solubility is the most important factor to improve its pharmacokinetics [3].

Hybrid curcumin molecules are currently used in medicinal chemistry to search for drugs aimed at treating multifactorial diseases. Compounds with radical scavenger properties of curcumin have shown remarkable activities related to the activation of the antioxidant response element (ARE). This controls the expression of genes, whose protein products have key roles in cellular defense against the toxicity of electrophiles and ROS, providing neuroprotective effects [4]. In addition, several compounds have shown significant inhibitory activities against α-glucosidase (α-Gls) [5]. Moreover, curcumin derivatives have attracted considerable attention in the search for compounds with antioxidant and antibacterial activities combined in one molecule, which have been proposed for chronic infections associated with epithelial damage by pulmonary stress [6]. The reported anticancer activities of modified curcumin, with varying structural features, such as aromatic side chains, di-keto functionality, active methylene groups, carbon linker chain, and mono-carbonyl analogs, show the high potential of these types of compounds [7]. Among them, the antitumor activity of curcumin derivatives against triple-negative breast cancer, a highly metastatic breast cancer cell line, was inversely correlated with their antioxidant activity, as measured by the DPPH assay [8].

Recently, anticancer therapies target the uncontrolled proliferation of cancer cells. However, these strategies have proved highly inefficient in treating metastatic solid tumors, since the mechanisms of migration are not inhibited by conventional anticancer drugs. Therefore, the search for compounds that inhibit migration and invasion of solid cancers is highly relevant [9,10,11]. In this context, the antimigratory activity of some curcumin derivatives has been reported [12,13].

A promising strategy for obtaining curcuminoids with ionic liquid characteristics can overcome the main drawbacks associated with curcumin. Ionic liquids are composed of bulky organic cations, in combination with a wide variety of anions, ranging from simple inorganic anions to more complex organic species [14], and have melting points below 100 °C [15]. Moreover, some ionic liquids contain nitrogen and those with anticancer properties have been reported. For example, imidazolium salts containing long alkyl chains exhibit high growth inhibition and variable cytotoxicity against a wide panel of cancer cell lines [16]. Di-pyridinium salts containing octyl chains have cytotoxic effects against glioma cells [17]. In addition, the synthesis of various heterocyclic curcumin analogs of pharmacological interest has been reported [18,19].

In this work, we have obtained new analogs of curcumin with ionic liquid characteristics, through the replacement of one phenyl group with an alkyl-substituted pyridinium group. The in vitro antioxidant properties and computational calculations allowed for insight into the structural–antioxidant properties relationship in these series of compounds. To assess the anticancer potential of these compounds, cell viability and cell migration assays were performed for human colon cancer (HT29), human breast cancer (MCF7) cells, in addition to NIH3T3 murine fibroblast, as a model of non-tumoral cell type. 

## 2. Materials and Methods

### 2.1. Chemical Synthesis 

**Scheme 1:**
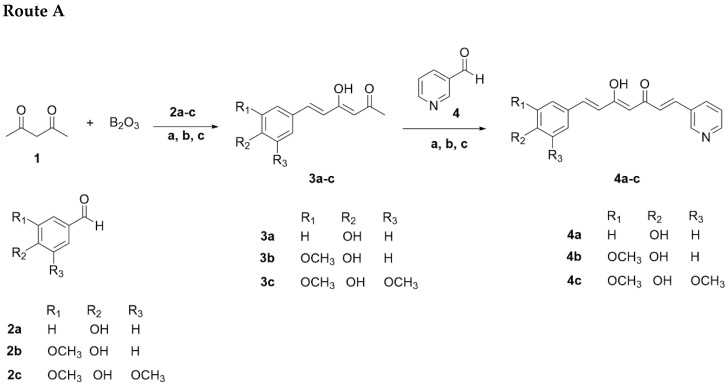

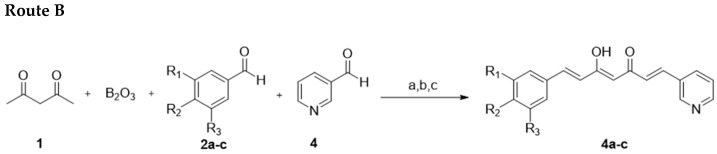
Synthesis of different pyridine curcuminoids 4a-c. Reagents and conditions: (a) B(n-BuO)_3_; (b) n-BuNH_2_, 80 °C, refluxed for 1 h; (c) HCl, 80 °C.

**Scheme 2:**
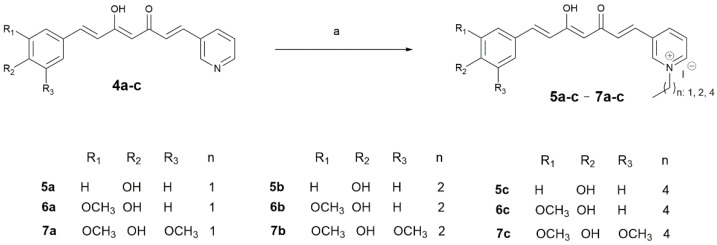
Synthesis of curcuminoid-derived pyridinium salts **5a-c**–**7a-c**. Reagents and conditions: (a) **4a-c** (1 equivalent), alkyl (ethyl, propyl, and pentyl) iodide (1 equivalent), DMF, MW, 200 W, 5 min.

All of the reagents were purchased from Merck or Sigma-Aldrich (St. Louis, MO, USA) and used without further purification. The solvents used for reactions, such as dichloromethane, ethyl acetate, hexane, and dimethylformamide were of HPLC grade ≥ 99.7%. The synthetic plan for the construction of the curcumin-analogs pyridinium salts is described in Scheme 1 and Scheme 2.

#### 2.1.1. Route A

Initially, aldehyde (**2a-c**) (9 mmol) and tri-n-butyl borate (20 mmol) in ethyl acetate (5 mL) were added to a stirring suspension of acetylacetone (10 mmol) and boric anhydride (10 mmol) in ethyl acetate (50 mL), which was vigorously stirred for 30 min at 80 °C. The reaction mixture was stirred at 80 °C for an additional 30 min, and then a solution of n-butylamine (4 mmol in 1.0 mL of EtOAc) was added dropwise over 15 min. The mixture was maintained at 80 °C for 8 h, cooled to room temperature, acidified with 0.5 N HCl (30 mL), and stirred at 80 °C for 30 min. The organic layer was separated and the aqueous layer was extracted with ethyl acetate (3 × 30 mL). The combined organic fractions were sequentially washed with saturated aqueous NaHCO_3_ and brine, dried over anhydrous Na_2_SO_4_, filtered, and concentrated under reduced pressure. The crude residue was purified by flash column chromatography (L × D.I × D.E: 70 cm × 24 mm × 28 mm) using a mixture of hexane/ethyl acetate 7:3 as eluent, followed by crystallization from a suitable solvent to obtain the monoaryl curcuminoids (**3a-c**). Following the same procedure, these compounds were used to obtain the pyridine curcuminoids **4a-c** by reaction with nicotinaldehyde (**4**) in a 1:1 molar ratio. The pyridine curcuminoids **4a-c** were obtained in yield ranges of 1.0–6.8% (Scheme 1, Table 1).

**Table 1:** Yields of different pyridine curcuminoid 4a-c.

	R_1_	R_2_	R_3_	Yield Route A	Yield Route B
**4a**	H	OH	H	<1.0	19.5
**4b**	OCH_3_	OH	H	6.8	20.2
**4c**	OCH_3_	OH	OCH_3_	5.0	20.8

#### 2.1.2. Route B “Mixed Pabon Reaction”

Briefly, aldehyde (**2a-c**) (10 mmol), nicotinaldehide (**4**) (10 mmol), and tri-n-butyl borate (20 mmol) dissolved in ethyl acetate (5 mL) were added to a stirring suspension of acetylacetone (10 mmol) and boric anhydride (10 mmol) in ethyl acetate (50 mL), which was vigorously stirred for 30 min at 80 °C. The reaction mixture was stirred at 80 °C for additional 30 min, then a solution of n-butylamine (4 mmol in 1.0 mL of EtOAc) was added dropwise over 15 min. The mixture was maintained at 80 °C for 8 h, cooled to room temperature, acidified with 0.5 N HCl (30 mL), and stirred at 80 °C for an additional 30 min. The organic phase was separated, and the aqueous layer was extracted with EtOAc (3 × 30 mL). The combined organic fractions were sequentially washed with saturated aqueous NaHCO_3_ and brine, dried over anhydrous Na_2_SO_4_, filtered, and concentrated under reduced pressure. The crude residue was purified by countercurrent chromatography (CCC). The solvent system selected for the purification was composed of hexane–ethyl acetate–methanol–water (5:5:5:5 *v/v/v/v*), acidified with 0.1% trifluoroacetic acid. In addition, the mobile phase operated at a flow rate of 4 mL/min in a head-to-tail mode, at a revolution speed of 700 rpm. The pyridine-curcuminoids **4a-c** were obtained in yield ranges of 19.5–20.8% (Scheme 1, Table 1).

#### 2.1.3. Curcuminoid-Derived Pyridinium Salts 

The quaternization reaction was performed by the reaction of pyridine curcuminoids (**4a-c**) (1 mmol) with alkyl bromides (ethyl, propyl, and pentyl iodides) (1 mmol) in dimethylformamide (DMF), using microwave irradiation (250 MW) at 80 °C for 10 min (Scheme 2). The end of the reaction was marked by adding water (1 mL) to the reaction mixture, resulting in the formation of an oily precipitate, which was subsequently washed with diethyl ether (4 × 2 mL) and separated by decantation. In addition, some of these salts were purified by CC using cellulose as stationary phase and 6:4 ethyl acetate–methanol as mobile phase. Finally, the purified compounds were maintained under a high vacuum for 4 h to obtain alkyl pyridinium salts (**5a-c**–**7a-c**). These salts were obtained in the form of creamy solids with yield ranges of 6.2–34.3% (Table 2).

**Table 2:** Yields of curcuminoid-derived pyridinium salts **5a-c**–**7a-c**.

Pyridinium Salt		Yield
**5**	**a**, n: 1	11.1
	**b**, n: 2	19.3
	**c**, n: 4	16.4
**6**	**a**, n: 1	34.0
	**b**, n: 2	25.1
	**c**, n: 4	6.2
**7**	**a**, n: 1	34.3
	**b**, n: 2	30.7
	**c**, n: 4	27.3

(1E,4Z,6E)-5-hydroxy-7-(4-hydroxyphenyl)-1-(pyridine-3-yl)-hepta-1,4,6-trien-3-one (**4a**). ^1^H NMR (400 MHz, DMSO-d6) δ: 8.84 (s, 1H), 8.55 (d, *J* = 4.4 Hz, 1H), 8.12 (d, *J* = 7.5 Hz, 2H), 7.54 (d, *J* = 8.2 Hz, 4H), 7.44 (dd, *J* = 7.7, 4.8 Hz, 1H), 6.81 (d, *J* = 8.3 Hz, 3H), 6.73 (dd, *J* = 16.7, 8.1 Hz, 2H), 6.11–5.81 (m, 1H). These spectroscopic data correspond to the previously reported data [20].

(1E,4Z,6E)-5-hydroxy-7-(4-hydroxy-3-methoxyphenyl)-1-(pyridine-3-yl)-hepta-1,4,6-trien-3-one (**4b**). ^1^H NMR (400 MHz, DMSO-d6) δ: 9.21 (s, 1H), 8.81 (dd, *J* = 18.1, 6.8 Hz, 2H), 8.00 (dd, *J* = 7.9, 5.7 Hz, 1H), 7.69 (d, *J* = 16.1 Hz, 1H), 7.57 (d, *J* = 15.8 Hz, 1H), 7.27 (d, *J* = 16.1 Hz, 1H), 7.20 (s, 1H), 7.15 (d, *J* = 8.3 Hz, 1H), 6.96 (d, *J* = 8.4 Hz, 1H), 6.72 (d, *J* = 15.8 Hz, 1H), 6.19 (s, 1H), 3.81 (s, 3H).These spectroscopic data correspond to the previously reported data [21].

(1E,4Z,6E)-5-hydroxy-7-(4-hydroxy-3,5-dimethoxyphenyl)-1-(pyridine-3-yl)-hepta-1,4,6-trien-3-one (**4c**). ^1^H NMR (400 MHz, DMSO-d6) δ: 9.10 (s, 1H), 8.87 (s, 1H), 8.57 (d, *J* = 4.6 Hz, 1H), 8.15 (d, *J* = 8.0 Hz, 1H), 7.62 (dd, *J* = 15.9, 6.6 Hz, 2H), 7.46 (dd, *J* = 7.9, 4.8 Hz, 1H), 7.06 (s, 2H), 7.03 (d, *J* = 5.0 Hz, 1H), 6.86 (d, *J* = 15.8 Hz, 1H), 6.14 (s, 1H), 3.83 (s, 6H). ^13^C NMR (101 MHz, DMSO) δ: 185.58, 180.81, 150.73, 149.86, 148.34, 142.44, 138.86, 136.24, 134.72, 130.86, 126.36, 125.23, 124.28, 121.69, 106.60, 101.94, 56.31, 39.52. HRMS (ESI) *m/z* calculated for C_20_H_19_NO_5_ [M]^+^; 353.1263, found 353.1263.

1-ethyl-3-((1E,4Z,6E)-5-hydroxy-7-(4-hydroxyphenyl)-3-oxohepta-1,4,6-trien-1-yl)-pyridin-1-ium iodide (**5a**). ^1^H NMR (400 MHz, methanol-d4) δ: 9.41 (s, 1H), 8.98 (d, *J* = 8.0 Hz, 1H), 8.87 (d, *J* = 8.0 Hz, 1H), 8.15 (m, 1H), 8.17 (m, 1H) 7.71 (m, 1H), 7.56 (dd, *J* = 17.4 Hz, 9.7 Hz, 2H), 6.86 (m, 2H), 7.32 (d, *J* = 16.0 Hz, 1H), 7.0 (d, *J* = 16.0, 1H), 6.74 (d, *J* = 16.0 Hz, 1H), 4.71 (m, 2H), 1.24–0.76 (m, 3H). HRMS (ESI) *m/z* calculated for C_20_H_20_NO_3_^+^ [M-I]^+^; 322.1438, found 322.1452.

3-((1E,4Z,6E)-5-hydroxy-7-(4-hydroxyphenyl)-3-oxohepta-1,4,6-trien-1-yl)-1-propylpyridin-1-ium iodide (**5b**). ^1^H NMR (400 MHz, methanol-d4) δ: 9.41 (s, 1H), 8.99 (d, *J* = 8.0 Hz, 1H), 8.88 (d, *J* = 8.0 Hz, 1H), 8.25 (m, 1H), 8.17 (m, 1H) 7.72 (m, 1H), 7.56 (dd, *J* = 17.4 Hz, 9.7 Hz, 2H), 6.86 (m, 2H), 7.32 (d, *J* = 16.0 Hz, 1H), 7.01 (d, *J* = 16.0, 1H), 6.74 (d, *J* = 16.0 Hz, 1H), 4.71 (m, 2H), 1.69 (p, *J* = 7.6 Hz, 1H), 1.48 (dq, J = 14.9, 7.3 Hz, 1H), 1.24–0.76 (m, 3H). HRMS (ESI) *m/z* calculated for C_21_H_22_NO_3_^+^ [M-I]^+^; 336.1594, found 336.1590.

3-((1E,4Z,6E)-5-hydroxy-7-(4-hydroxyphenyl)-3-oxohepta-1,4,6-trien-1-yl)-1-pentylpyridin-1-ium iodide (**5c**). ^1^H NMR (400 MHz, methanol-d4) δ: 9.41 (s, 1H), 8.99 (d, *J* = 4.0 Hz, 1H), 8.87 (d, *J* = 4.0 Hz, 1H), 8.14 (m, 1H), 7.70 (d, *J* = 16.0 Hz, 2H), 7.57 (d, *J* = 8.0 Hz, 2H), 7.32 (d, *J* = 16.0 Hz, 1H), 6.87 (d, *J* = 8.0, 3H), 6.71 (d, *J* = 16.0 Hz, 1H), 4.70 (t, *J* = 7.5 Hz, 2H), 2.28–1.92 (m, 2H), 1.44 (s, 4H), 1.04–0.80 (m, 3H). HRMS (ESI) *m/z* calculated for C_23_H_26_NO_3_^+^ [M-I]^+^; 364.1907, found 364.1917.

1-ethyl-3-((1E,4Z,6E)-5-hydroxy-7-(4-hydroxy-3-methoxyphenyl)-3-oxohepta-1,4,6-trien-1-yl)-pyridin-1-ium iodide (**6a**). ^1^H NMR (400 MHz, DMSO-d6) δ: 9.40 (s, 1H), 9.18 (s, 1H), 8.97 (d, *J* = 5.9 Hz, 1H), 8.80 (d, *J* = 8.2 Hz, 2H), 8.11 (dd, *J* = 8.0, 6.2 Hz, 1H), 7.59 (d, *J* = 16.0 Hz, 1H), 7.52 (d, *J* = 15.8 Hz, 1H), 7.24 (d, *J* = 16.0 Hz, 1H), 7.09 (s, 1H), 6.91 (d, *J* = 8.8 Hz, 1H), 6.70 (d, *J* = 15.8 Hz, 1H), 6.15 (s, 1H), 4.56 (q, *J* = 7.2 Hz, 2H), 1.51 (t, *J* = 7.3 Hz, 3H). HRMS (ESI) *m/z* calculated for C_21_H_22_NO_4_^+^ [M-I]^+^; 352.1543, found 352.1547.

3-((1E,4Z,6E)-5-hydroxy-7-(4-hydroxy-3-methoxyphenyl)-3-oxohepta-1,4,6-trien-1-yl)-1-propylpyridin-1-ium iodide (**6b**). ^1^H NMR (400 MHz, DMSO-d6) δ: 9.43 (s, 1H), 8.94 (dd, *J* = 6.8 Hz, 3H), 8.26–8.12 (m, 1H), 7.64 (dd, *J* = 15.8, 3.6 Hz, 2H), 7.34 (s, 1H), 7.28 (d, *J* = 16.0 Hz, 1H), 7.18 (d, *J* = 8.1 Hz, 1H), 6.83 (dd, *J* = 19.5, 12.0 Hz, 2H), 6.16 (s, 1H), 4.54 (t, *J* = 7.0 Hz, 2H), 3.81 (s, 3H), 1.97 (dq, *J* = 14.1, 7.1 Hz, 2H), 0.89 (t, *J* = 7.2 Hz, 3H). HRMS (ESI) *m/z* calculated for C_22_H_24_NO_4_^+^ [M-I]^+^; 366.1700, found 366.1705.

3-((1E,4Z,6E)-5-hydroxy-7-(4-hydroxy-3-methoxyphenyl)-3-oxohepta-1,4,6-trien-1-yl)-1-pentylpyridin-1-ium iodide (**6c**). ^1^H NMR (400 MHz, DMSO-d6) δ: 9.76 (s, 1H), 9.60 (s, 1H), 9.10 (d, *J* = 5.6 Hz, 1H), 8.92 (d, *J* = 7.9 Hz, 1H), 8.20 (t, *J* = 6.9 Hz, 1H), 7.66 (dd, *J* = 15.8, 6.1 Hz, 2H), 7.37 (t, *J* = 7.9 Hz, 2H), 7.19 (d, *J* = 8.0 Hz, 1H), 6.94–6.80 (m, 2H), 6.20 (s, 1H), 4.63 (t, *J* = 7.0 Hz, 2H), 3.84 (s, 3H), 2.09–1.84 (m, 2H), 1.44–1.11 (m, 4H), 0.88 (t, *J* = 6.6 Hz, 3H). HRMS (ESI) *m/z* calculated for C_24_H_28_NO_4_^+^ [M-I]^+^; 394.2013, found 394.2016.

1-ethyl-3-((1E,4Z,6E)-5-hydroxy-7-(4-hydroxy-3,5-dimethoxyphenyl)-3-oxohepta-1,4,6-trien-1-yl)-pyridin-1-ium iodide (**7a**). ^1^H NMR (400 MHz, DMSO-d6) δ: 9.57 (s, 1H), 9.09 (d, *J* = 5.6 Hz, 1H), 8.90 (d, *J* = 7.7 Hz, 1H), 8.28–8.11 (m, 1H), 7.78–7.55 (m, 2H), 7.36 (d, *J* = 18.0 Hz, 1H), 7.09 (s, 2H), 6.95 (d, *J* = 15.9 Hz, 1H), 6.21 (s, 1H), 4.66 (q, *J* = 6.9 Hz, 2H), 3.83 (s, 6H), 1.59 (t, *J* = 7.2 Hz, 3H). ^13^C NMR (101 MHz, DMSO) δ: 187.26, 177.80, 174.18, 148.18, 144.03, 143.33, 142.67, 139.00, 135.26, 131.57, 130.44, 128.03, 124.93, 121.61, 119.63, 106.78, 102.88, 59.53, 56.77, 56.26, 16.15. HRMS (ESI) *m/z* calculated for C_22_H_24_NO_5_^+^ [M-I] ^+^; 382.1649, found 382.1658.

3-((1E,4Z,6E)-5-hydroxy-7-(4-hydroxy-3,5-dimethoxyphenyl)-3-oxohepta-1,4,6-trien-1-yl)-1-propylpyridin-1-ium iodide (**7b**). ^1^H NMR (400 MHz, DMSO-d6) δ: 9.50 (s, 1H), 9.14 (s, 1H), 9.05 (d, *J* = 6.0 Hz, 1H), 8.92 (d, *J* = 8.2 Hz, 1H), 8.30–8.12 (m, 1H), 7.67 (dd, *J* = 15.9, 3.3 Hz, 2H), 7.32 (d, *J* = 16.0 Hz, 1H), 7.09 (s, 2H), 6.94 (d, *J* = 15.8 Hz, 1H), 6.20 (s, 1H), 4.58 (t, *J* = 7.3 Hz, 2H), 3.83 (s, 6H), 2.00 (h, *J* = 7.3 Hz, 2H), 0.92 (t, *J* = 7.3 Hz, 3H). ^13^C NMR (101 MHz, DMSO) δ: 187.26, 177.80, 174.18, 148.18, 144.03, 143.33, 142.67, 139.00, 135.26, 131.57, 130.44, 128.03, 124.93, 121.61, 119.63, 106.78, 102.88, 59.53, 56.77, 56.26, 16.15. HRMS (ESI) *m/z* calculated for C_23_H_26_NO_5_^+^ [M-I]^+^; 396.1805, found 396.1815.

3-((1E,4Z,6E)-5-hydroxy-7-(4-hydroxy-3,5-dimethoxyphenyl)-3-oxohepta-1,4,6-trien-1-yl)-1-pentylpyridin-1-ium iodide (**7c**). ^1^H NMR (400 MHz, DMSO-d6) δ: 9.50 (s, 1H), 9.14 (s, 1H), 9.05 (d, *J* = 6.0 Hz, 1H), 8.92 (d, *J* = 8.2 Hz, 1H), 8.30–8.12 (m, 1H), 7.67 (dd, *J* = 15.9, 3.3 Hz, 2H), 7.32 (d, *J* = 16.0 Hz, 1H), 7.09 (s, 2H), 6.94 (d, *J* = 15.8 Hz, 1H), 6.20 (s, 1H), 4.58 (t, *J* = 7.3 Hz, 2H), 3.83 (s, 6H), 2.00 (h, *J* = 7.3 Hz, 2H), 0.92 (t, *J* = 7.3 Hz, 3H). ^13^C NMR (101 MHz, DMSO) δ: 187.22, 177.71, 148.13, 144.17, 143.31, 142.66, 138.99, 135.23, 131.51, 130.40, 127.98, 124.86, 121.55, 106.76, 102.78, 61.11, 56.20, 30.14, 27.53, 21.57, 13.73. HRMS (ESI) *m/z* calculated for C_25_H_30_NO_5_^+^ [M-I]^+^; 424.2118, found 424.2121.

### 2.2. GC–MS Analysis

GC–MS analysis was performed with Trace 1300 gas chromatograph coupled to ISQ mass spectrometer (Thermo Fisher Scientific 713100331, ISQ 130729 2215, Grand Avenue Parkway Autin, TX, USA) series equipment equipped with an Rtx-5MS w/integra-guard capillary column (Crossbond 5% diphenyl–95% dimethyl polysiloxane) 30 m in length, 0.25 mm internal diameter, and 0.25 mm thick. Briefly, 3 µL of the sample at a temperature of 250 °C were injected into the injection port under splitless mode with the following conditions: 60 °C with a ramp of 10 °C/min to 300, waiting time of 5 min, drag flow of 1.5 mL/min. All of the assays were performed in scan mode.

### 2.3. Computational Calculations

In the present work, density functional theory (DFT) calculations were carried out at the M06-2X/6-311+G(d,p) level, using the Gaussian 09 software [22]. No imaginary vibrational frequencies were found at the optimized geometries, indicating that they are the true minimal of the potential energy surface. Solvent effects were taken into account by the polarizable continuum model (PCM), using parameters for water [23]. The formation of free radical species from phenols, and then their radical scavenging properties, occur in the biological systems mainly by the hydrogen atom transfer (HAT) mechanism, which is governed by the homolytic bond dissociation enthalpy (BDE) [24]. Therefore, we calculated the BDE of O–H bonds in our molecules, as follows:

H–O → H^•^ + O^•     ^ BDE = H*(*H^•^) + H*(*O^•^)–H*(*O–H)

where H(H^•^), H(O^•^), and H(O–H) are the corresponding enthalpies. The lower the BDE values, the easier the dissociation of the O–H bond, and the easier the radical is formed. 

### 2.4. In Vitro Antioxidant Activity 

#### 2.4.1. DPPH Radical Scavenging Method

The 1,1-diphenyl-2-picrylhydrazyl (DPPH) assay was performed according to the microwell-adopted procedure described by Brand-Williams et al., 1995 [25]. Briefly, a stock solution of DPPH (20 mg/L) in methanol was prepared, then 75 μL of the compounds or control (80% methanol) were mixed with 150 μL of DPPH reagent (several dilutions of the extracts 1:2 were carried out using methanol). After 15 min, the absorbance was measured at 515 nm using a microplate reader spectrophotometer. The results were expressed as the half-maximal inhibitory concentration (IC_50_, µM) by a mean of three determinations, in which the percentage of antioxidant activity was first calculated for each dilution as follows:

% Scavenging DPPH free radical = 100 × (1 − AE/AD)

where AE is the absorbance of the solution after adding the IL and AD is the absorbance of DPPH solution. Then, the half-maximal (IC_50_) inhibitory concentration value was calculated in mg/ml by the linear regression analysis, and the results were expressed in µM using the MW of each compound.

#### 2.4.2. Ferric Reducing Antioxidant Power (FRAP) Assay

The FRAP assay was performed according to the procedure described by Benzie and Strain 1996 [26]. The reagent was prepared by mixing 25 mL of 300 mM of acetate buffer (pH 3.6) and 2.5 mL of 2,4,6-tris(2-pyridyl)-s-triazine in 40 mM of HCl and 2.5 mL of 20 mM FeCl_3_·6H_2_O. Then, 0.05 mL of the compounds or standard (FeSO_4_·7H_2_O, Fe^2+^ concentration vs. absorbance) were added to 1.5 mL of FRAP solution and 0.15 mL of water. Thereafter, the mixture was incubated at room temperature for 15 min. The final results of the product (ferrous tripyridyl triazine complex) were read at a wavelength of 593 nm, using a spectrophotometer. The percentage of Fe^3+^ scavenging (reduction to Fe^2+^) was calculated by comparison with the standard curve.

### 2.5. Cell Lines and Culture

Three cell lines were used to assess the antitumor activities of the compounds studied in this work: Human colon cancer-derived HT29, human breast cancer-derived cells MCF7, and murine fibroblast NIH3T3. The cell lines were cultured in Dulbecco’s Modified Eagle’s Medium (GIBCO) supplemented with bovine calf serum to a final concentration of 10%, glucose (4500 mg/L), L-glutamine (2 mM), penicillin (50 U/L), and streptomycin (50 µg/mL). The cultures were incubated at 37 °C and 5% CO_2_ in a humidified atmosphere. 

#### 2.5.1. Viability Assay

Viability was assessed using a tetrazolium salt, the 3-(4,5-dimethylthiazol-2-yl)-2,5-diphenyl tetrazolium bromide (MTT) (Cayman Chemical Company, Ann Arbor, MI, USA) [27]. For this assay, 1.5 × 10^4^ cells per well were seeded in a 96-well plate and incubated for 24 h with different concentrations of the curcumin-derived compounds (10, 20, and 60 μM dissolved in water with 0.5% DMSO). In addition, DMSO was included as a vehicle control at 0.5%. Following this treatment, the culture medium of the cells was changed and the MTT reagent was added and incubated for 3 h. Then, cells were lysed and the crystals formed were dissolved in isopropanol (Cayman Chemical Company), for reading in a multiplate reader (Thermo Scientific, Waltham, MA, USA) at 570 nm. The results are expressed as a percentage of vehicle control.

#### 2.5.2. Fluorescence Images 

MCF7 cells were seeded at 50–60% confluence on coverslips in a 12-well plate. Then, the cells were treated with 20 µM of compounds, incubated for 1 h, and washed twice with phosphate-buffered saline to remove the compounds. Images were digitally acquired with an Olympus BX53 fluorescence microscope (Olympus) coupled to a CCD camera. Digital images were taken using Q-Capture Pro 7 software (Qimagine, Inc. Surrey, BC, Canada).

#### 2.5.3. Cell Migration Assay

Cells from different cell lines (HT29, MCF7 or NIH3T3) were seeded at 70–80% confluence in polystyrene plates of 60 mm. A vertical scratch was made on the plate using a micropipette tip of 200 μL. Then, the cells were washed twice with phosphate-buffered saline to remove the non-adhered cells. Thereafter, 20 μM of compounds were added in addition to a control plate with only 0.5% DMSO as a vehicle and the plates were incubated for 16 h. Photographs of the scratch were taken before adding the extracts and after incubation with a Canon^®^ Model Eos Rebel XSI camera. Migration was determined by measuring the delta of the area of wound closure (0–16 h area) obtained by the ImageJ computer program. The results were expressed as the relative migration rate within 16 h, which is the percentage of migration to the vehicle control in that period (control: 100% of migration/16 h). 

#### 2.5.4. Statistical Analysis

For statistical analysis, the results represent measurements of at least three independent experiments. The data from the groups were compared using the analysis of variance with Tukey’s post hoc test. A *p*-value < 0.05 was considered as statistically significant. The GraphPad Prism 5 software program (San Diego, CA, USA) was used and data were expressed as the mean ± standard deviation (SD).

## 3. Results and Discussion

### 3.1. Chemical Synthesis 

As shown in Scheme 1 and Scheme 2, the synthesis of curcumin-analog alkyl pyridinium salts was achieved with a total of three reaction steps, according to the Pabon method [28]. At first, phenyl diketones **3a**-**e** were obtained by the reaction of 2,4-pentadione 1, boron trioxide (B_2_O_3_) at reflux in a 1:1 molar ratio, 0.9 equivalent of aldehyde **2a**-**e**, 2 equivalent of tributyl borate (B(n-BuO)_3_), and butylamine (0.4 M in AcOEt). Column chromatography (CC) using hexane–ethyl acetate mixtures in 8:2, 7:3, 6:4, and 1:1 as eluents allowed us to obtain the phenyl diketones **3a**-**e,** respectively as yellow solids and 13.4–49.0% reaction yields. To obtain the curcuminoids **4a**-**c**, a second Pabon condensation, using the same procedure, was carried out, maintaining an equivalent molar ratio of 1:1 between the previously synthesized phenyl diketones **3a**-**e** and the nicotinaldehyde **4** (Route A). The low yields revealed by TLC and gas chromatography (GC–MS), indicating low consumption of the starting reagents, led us to explore the effect of some changes in the reaction and purification methodologies. Curcuminoids **4a**-**c**, which were performed by CC using petroleum ether–ethyl acetate mixtures in 7:3, 6:4, 1:1, 4:6, and 3:7, obtained **4a**-**c** as orange solids. A “one-pot” process, involving the double-condensation of two aldehydes of different nature in a single synthetic step (Route B), named by us as “mixed Pabon reaction”, allowed for the reduction of reaction times and purification processes (Scheme 1). In addition, these changes improved two fundamental parameters of green chemistry, namely, the atom economy (EA) and atom efficiency (EfA). For example, the linear synthesis of pyridine curcuminoid **4c** led to EA and EfA values of 54.03 and 2.0, while the mixed Pabon reaction led to values of 90.66 and 19, respectively. The yields obtained by this procedure, for the **4a**-**c** series were in the range of 19.5–20.8%, and the purification was efficiently carried out by countercurrent chromatography (CCC).

CCC is a separation technique with several advantages, including the lack of solid support, which could result in irreversible adsorption of target compounds [29]. For example, the solvent system selected for the purification of **4b** was composed of 5:5:5:5 HEMW at *v/v/v/v* (hexane–ethyl acetate–methanol-water), which provided a KD value of 1.72 for compound **4b** with Rf 0.9 and 0.74 for by-product **4b**. A similar solvent system was used for the separation of curcuminoids from curcumin and turmeric powder [30]. 

Finally, the reaction of curcuminoids **4a**-**c** with alkyl iodides (ethyl, propyl, and pentyl) to obtain the alkyl pyridinium salts, in dimethylformamide (DMF) and a 1:1 molar ratio, was carried out under 10 min of microwave irradiation (MW), as previously described [31,32] (Scheme 2). Some of these salts were purified by CC using cellulose as the stationary phase and 6:4 mixtures of ethyl acetate/methanol as eluent, obtaining yield ranges of 6.2–34.3%. 

### 3.2. In Vitro and In Silico Assessment of Antioxidant Capacity 

The in vitro antioxidant capacity of compounds was evaluated through the DPPH and FRAP assays. In addition, computational analyses were performed for insight into the structure of compounds and to rationalize the experimental results. Table 3 shows the results for all of the synthetized compounds and curcumin, which were used as a standard to compare the antioxidant capacity.

Among the derivatives studied, **4a**-**c** curcumin analogs exhibited the highest DPPH quenching capacity, which is similar to or better than curcumin. Pyridinium salts **5a**-**c** and **6a**-**c** showed DPPH quenching capacities lower than curcumin. However, in the third group of pyridinium salts (**7a**-**c**) possessing two methoxy groups, the derivatives **7b** and **7c** showed higher DPPH quenching capacities than curcumin in the DPPH and FRAP assays.

Notably, the increase in the length of the N-alkyl chain has different effects on the antioxidant capacity for each group of salts. On the one hand, group **5** diminishes the antioxidant capacity, with an increase in the length of the chain. On the other hand, group **7** shows a drastic increase in the antioxidant capacity with an increase in the number of carbon atoms in the chain. Unlike groups **5** and **7**, group **6** does not show any tendency.

Moreover, the FRAP assay results showed that groups **5** and **6** have the lowest reducing power, with no significant influence with the change in the length of the N-alkyl chain. The group curcuminoids-pyridine **4a**-**c** presented higher values than groups **5** and **6**, but they were below the reducing power of curcumin. Group **7** showed a drastic drop with an increase in the number of carbon atoms in the chain. The highest values of reducing power, following the same tendency as DPPH in regards to the length of the alkyl chain, showed that **7c** is the curcuminoid pyridinium salt with the highest reducing power. Both assays showed that alkyl pyridiniums of group **7** (two methoxyl groups in phenyl ring) exhibit the best antioxidant capacity, which increases along with the length of the N-alkyl chain. 

To rationalize our results, bond dissociation enthalpy (BDE) calculations in gas- and aqueous phases were conducted. Initially, we studied non-ionic compounds **4a**-**c** as well as one ionic compound of each family **5a**, **6a**, and **7a**. For our calculation study, we selected all of the compounds of group **4** to examine the influence of methoxy groups in the phenyl ring. In particular, we selected the compounds that possessed the shortest N-alkyl chain of each group (**5a**, **6a**, and **7a**), considering that the length of the alkyl chain should not significantly affect the electronic density of OH bonds in the compounds.

**Figure 2:**
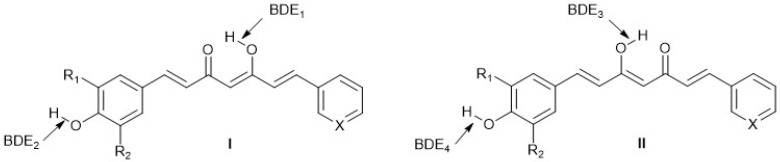
Identification of bond dissociation enthalpies (BDEs) in the possible tautomers (I and II) of the compounds studied.

Table 4 shows the BDE values for the hydroxyl groups of the compounds. For the calculation, we have considered both possible tautomers for the keto-enol equilibrium, as shown in Figure 2. Therefore, four BDEs were calculated for each one of the six compounds studied, both in gas phase and water as solvent.

For the compounds of group **4**, the results show that all of the O–H BDEs in the tautomer II were lower than their equivalents in tautomer I. Compounds **5a**, **6a**, and **7a** showed remarkably high values of BDE for tautomer II compared with I. Notably, water solvation has a different impact on the BDE of each O–H. For instance, for the tautomer I of the group **4** compounds, the solvation effect of water reduced the O–H BDEs values in the gas phase. However, the opposite effect occurs for their tautomers II, except for **4b**. The most significant effect observed is the remarkable reduction of the O–H BDE values when water solvation was considered for **5a**, **6a**, and **7a**. This indicates a relevant stabilization of the radicals formed in water compared with the gas phase, as seen for tautomer II of these ionic compounds. The lower values of BDEs in the water solution correspond to the structurally related curcuminoid-pyridine **4c** and the curcuminoid pyridinium salt **7a**, which is consistent with the obtained experimental results.

### 3.3. Preliminary Antitumor Evaluation

To evaluate the biological activity of the compounds on cells, an MTT assay was performed by incubating HT29, MCF7, and NIH3T3 cells in the presence of the compounds and vehicle control for 24 h. Although curcumin-derived compounds have been reported to exert an antiproliferative inhibitory effect in the range of 10–20 μM (IC_50_), we decided to test a wide range of concentrations to observe effects not only in proliferation and cell migration, but also in cell viability. The following reports illustrate the range of concentrations used in the antitumor assessment of related compounds [33,34,35]. This is especially important in our trials, since we test previously unreported compounds and use cell lines of diverse origins, such as colon cancer (HT29), breast cancer (MCF7), and mouse fibroblast (NIH3T3). 

**Figure 3:**
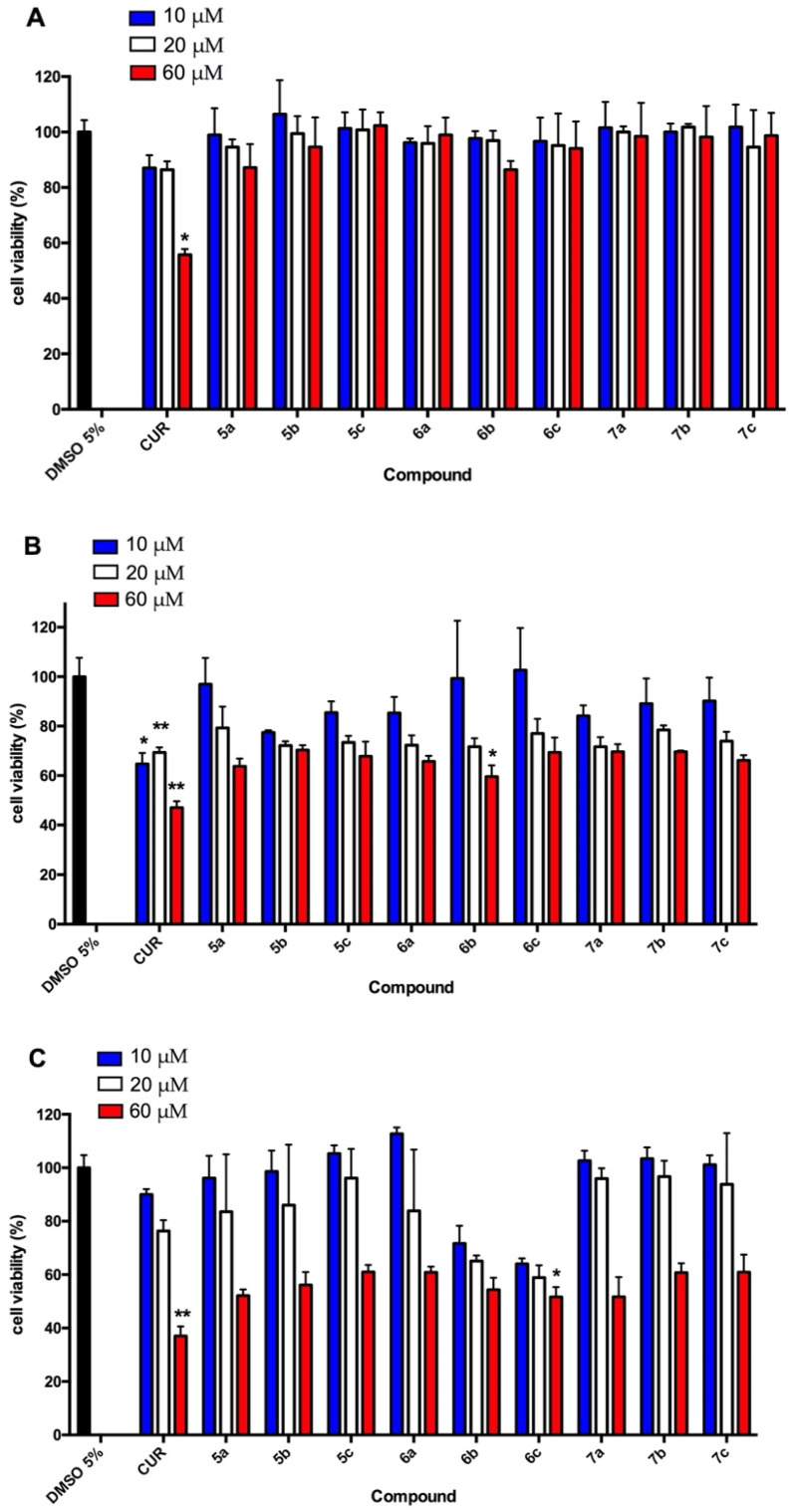
Effect of different concentrations of curcumin-analogs on the viability of two tumor cell lines: HT29 (**C**), MCF7 (**B**), and the non-tumoral NIH3T3 (**A**) cell line. Cells were incubated in the presence or not of the distinct compounds at three final concentrations (10, 20, and 60 µM) for 24 h. Then, an MTT assay was used to evaluate the cell viability. Results are expressed as mean ± SD of three independent experiments. The statistical significance shown is with respect to the untreated controls (DMSO 0.5%). * *p* < 0.05; ** *p* < 0.01.

As shown in Figure 3, a significant concentration-dependent decrease in the cell viability was observed in the tumoral cell lines when they were incubated with curcumin (CUR) as a reference compound. The effect was more noticeable over HT29. Notably, the viability of the non-tumoral cell line NIH3T3 was not significantly affected by CUR (*p* > 0.05).

All of the compounds produced a significant decrease in the viability of HT29 and MCF7 cells only at the highest concentration (60 µM). In general, a more noticeable effect over MCF7 is observed, since six of the nine compounds (**7a**, **7b**, **7c**, **5b**, **5c**, and **6a**) significantly decreased the cell viability at 10 or 20 µM. For HT29, the effects were significant only at 60 µM. For this cell line, compounds **6b** and **6c** showed the highest cytotoxic effects. Notably, all of the compounds showed no significant in vitro effect over the non-tumoral cell line NIH3T3, similar to CUR. However, none of them decrease viability in HT29 cells as effectively as CUR at the highest concentration (32.8 ± 6.4% viability), with compound **6c** as the more active (51.6 ± 3.8%). Related to MCF7, compound **6b** generates a very similar effect (60.0 ± 4.6%) to CUR (60.2 ± 1.5%) at 60 µM, but only CUR was found to be significantly active from 20 µM (68.1 ± 5.2%). 

**Figure 4:**
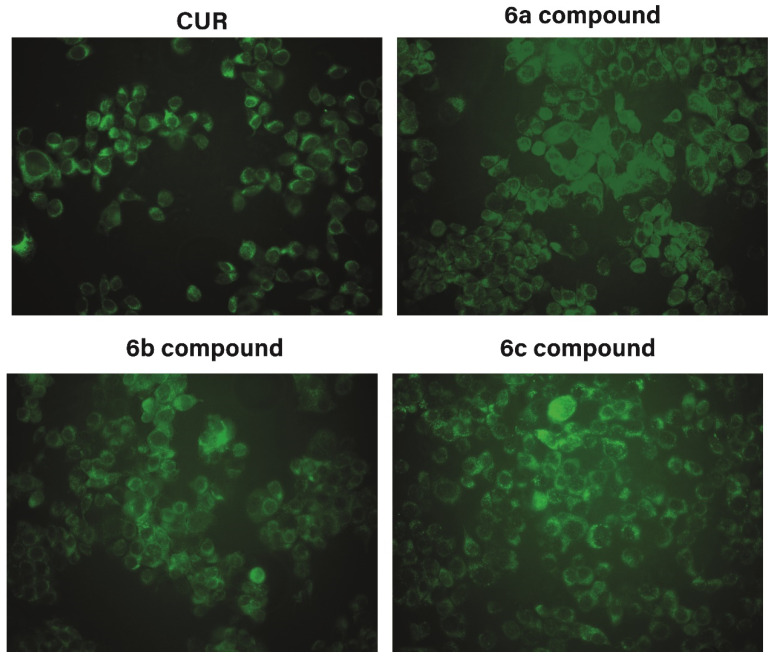
Incorporation of curcumin-derived compounds for the tumor cell line MCF7. The cells were incubated with the different curcumin-derived compounds **6a**-**c** with 20 µM for 1 h with curcumin as the control.

Moreover, to visualize the capacity of the derivates for incorporation inside the cell, we took advantage of fluorescence emission for these compounds. Series **6** (**6a**-**c**) were selected, specifically for their different results in the MCF7 cell line at 20 μM. Interestingly, all of the treatments showed a perinuclear distribution, and the three analyzed compounds of series **6** showed a major fluorescence compared with the control CUR. However, compound **6a** showed a higher signal compared with the other compounds (Figure 4).

Recently, the intracellular localization of curcumin and polysorbate-80 micelles of curcumin were studied in Caco-2 cells. The authors reported that after 30 min, curcumin from each formulation was associated with mitochondria and lysosomes. After 180 min, native curcumin was associated with mitochondria and peroxisomes, while micellar curcumin was associated only with peroxisomes. Interestingly, the subcellular localization was not associated with the differences in bioavailability in humans [36].

**Figure 5:**
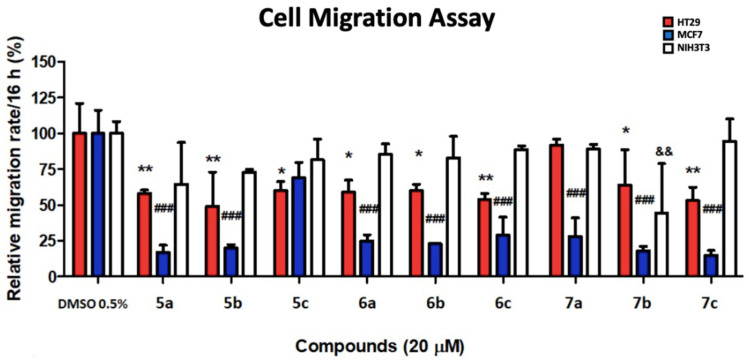
Effect of curcumin-derived compounds on the migration capacity of two tumor cell lines: HT29 and MCF7, and the non-tumoral NIH3T3 cell line. Cells were seeded in 24-well plates and a wound-healing assay was conducted in the presence of the distinct compounds (20 µM of final concentration). Microphotographs were taken at 0 and 24 h to calculate the percentage of cell-free area reduction, which is directly dependent on the in vitro cell migration. Results are expressed as the mean + SD of the relative migration rate in 16 h regarding the control (DMSO 0.5% as 100% of cell migration) and are representative of three independent experiments. The statistical significance shown is with respect to the controls (DMSO 0.5%), * *p* < 0.05; ** *p* < 0.01, for HT29; ^###^ *p* < 0.001 for MCF7, and ^&&^ *p* < 0.01 for NIH3T3 cells.

Cell migration is a very fundamental aspect of cancer since it is one of the initial steps of tumor metastasis [37]. We evaluated the effects of curcumin-analogs on the in vitro 2-D cell migration ability through a wound-healing assay at 20 µM of final concentration. The results showed a marked negative effect of CUR on cell migration in the three cell lines (Figure 5). Most of the 10 compounds significantly inhibited the in vitro tumor cell migration, except for compounds **7a** in HT29 and **5c** in MCF7. The strongest inhibition was observed for **7c** (53.0 ± 7.4% in HT29 and 15.0 ± 3.3% in MCF7 cell migration), but it was less than CUR (39.0 ± 12.5% in HT29 and 23.0 ± 5.2% in MCF7 cell migration). In general, a higher effect on in vitro cell migration was observed in MCF7 cells. Noteworthy, compound **6b** showed a good combination of effects, since it significantly reduced cell migration in both HT29 and MCF7 cells, but also showed a significant decrease in the cell viability at 20 µM (Figure 5), without affecting the non-tumoral NIH3T3 cell line: Neither in viability nor in migration, unlike the CUR compound.

## 4. Conclusions

In conclusion, using the two-step process described herein, it has been possible to obtain curcumin with ionic liquid characteristics. Initially, a “one-pot” reaction involving the double-condensation of two aldehydes of different nature in a single synthetic step, named by us as “mixed Pabon reaction”, allowed us to obtain pyridine curcumoids **4a**-**c**. Then, curcuminoid *N*-alkylpyridinium salts were obtained by microwave assisted synthesis in moderate global yields. The in vitro antioxidant properties demonstrated that group **4**, pyridine curcumoids, and group **7**, curcuminoid *N*-alkylpyridinium salts with two methoxy groups in phenyl ring, presented the best antioxidant capacity. For group **7**, an increase in the antioxidant capacity was observed as the length of the N-alkyl chain increased. We rationalized the experimental results by density functional theory (DFT) calculations of the bond dissociation enthalpy (BDE) for O–H in each compound. BDEs, obtained in the gas phase and water, showed a notable impact of water solvation on the stability of radicals. The lower values of BDEs in the water solution correspond to the structurally related curcuminoid-pyridine **4c** and the curcuminoid pyridinium salts **7a**, which is consistent with the experimental results. Additionally, a preliminary assessment of cell viability and cell migration assays showed that these compounds mainly cause inhibition of the cell migration observed in MCF7 cancer cells, without affecting the non-tumoral NIH3T3 cell line: Neither in viability nor in migration. Finally, these results suggest that some of these compounds could be selective antimetastatic agents, and we hope to carry out future studies to unveil the cellular mechanisms involved.

## Figures and Tables

**Table 3:** Antioxidant properties of the different curcuminoids synthesized.

Compound	DPPH (IC_50_, μM)	Mole Fe^2+^/Mole of Compound
**5a**	128.9 ± 0.11	0.6 ± 0.010
**5b**	184.2 ± 1.36	0.6 ± 0.004
**5c**	>190	0.6 ± 0.008
**6a**	>190	0.7 ± 0.004
**6b**	102.6 ± 0.45	0.9 ± 0.009
**6c**	>190	0.8 ± 0.056
**7a**	26.5 ± 0.59	2.3 ± 0.097
**7b**	5.7 ± 0.36	3.8 ± 0.012
**7c**	1.8 ± 0.22	7.3 ± 0.242
**4a**	1.33 ± 0.10	1.0 ± 0.037
**4b**	14.2 ± 0.34	2.1 ± 0.103
**4c**	0.90 ± 0.07	1.6 ± 0.008
**CUR**	7.0 ± 0.60	2.7 ± 0.050

**Table 4:** Bond dissociation enthalpies (kcal/mol) for hydroxyls in compounds, both in gas phase (g) and water as solvent (w).

Tautomer	Compound	4a	4b	4c	5a	6a	7a
I	BDE1 (g)	109.34	100.32	100.34	100.16	100.16	100.17
	BDE1 (w)	96.9	97.18	97.09	109.47	96.93	96.91
	BDE2 (g)	109.37	100.2	100.38	100.16	100.14	100.17
	BDE2 (w)	96.84	97.07	97.08	109.26	96.81	96.7
II	BDE3 (g)	84.74	84.5	80.55	269.77	296.41	316.61
	BDE3 (w)	85.97	82.9	80.88	86.23	83.11	81.29
	BDE4 (g)	84.19	84.1	80.22	269.26	296.08	316.61
	BDE4 (w)	85.24	82.41	80.63	85.49	82.68	80.81

## Data Availability

All of the data is contained within the article.
